# Evidence that vitronectin is a potent migration-enhancing factor for cancer cells chaperoned by fibrinogen: a novel view of the metastasis of cancer cells to low-fibrinogen lymphatics and body cavities

**DOI:** 10.18632/oncotarget.12003

**Published:** 2016-09-13

**Authors:** Gabriela Schneider, Ewa Bryndza, Agata Poniewierska-Baran, Karol Serwin, Malwina Suszynska, Zachariah P. Sellers, Michael L. Merchant, Alagammai Kaliappan, Janina Ratajczak, Magda Kucia, Nichola C. Garbett, Mariusz Z. Ratajczak

**Affiliations:** ^1^ Stem Cell Institute at the James Graham Brown Cancer Center, University of Louisville, KY, USA; ^2^ James Graham Brown Cancer Center, Department of Medicine, University of Louisville, KY, USA; ^3^ Kidney Disease Program, Department of Medicine, University of Louisville, KY, USA; ^4^ Department of Regenerative Medicine, Medical University of Warsaw, Poland; ^5^ Department of Physiology, Pomeranian Medical University, Szczecin, Poland

**Keywords:** vitronectin, urokinase plasminogen activator receptor (uPAR), fibrinogen, cancer metastasis, chemotaxis

## Abstract

Diluted (1%) plasma induces migration of malignant cell lines much more strongly than potent pro-metastatic factors. To characterize the factor(s) present in diluted plasma responsible for this phenomenon we performed i) heat inactivation, ii) dialysis, iii) proteinase K treatment, and iv) molecular size filtration studies. We found that this remarkable pro-migratory activity of diluted normal plasma is associated with a ~50–100-kD protein that interacts with G_αI_ protein-coupled receptors and activates p42/44 MAPK and AKT signaling in target cells. Since this pro-migratory activity of 1% plasma decreases at higher plasma concentrations (> 20%), but is retained in serum, we hypothesized that fibrinogen may be involved as a chaperone of the protein(s). To identify the pro-migratory protein(s) present in diluted plasma and fibrinogen-depleted serum, we performed gel filtration and hydrophobic interaction chromatography followed by mass spectrometry analysis. We identified several putative protein candidates that were further tested in *in vitro* experiments. We found that this pro-migratory factor chaperoned by fibrinogen is vitronectin, which activates uPAR, and that this effect can be inhibited by fibrinogen. These results provide a novel mechanism for the metastasis of cancer cells to lymphatics and body cavities, in which the concentration of fibrinogen is low, and thus suggests that free vitronectin stimulates migration of tumor cells.

## INTRODUCTION

A major obstacle in cancer therapy is the tendency of cancerous cells to leave a primary tumor and metastasize to different vital organs. However, the mechanisms that govern this process are still not well understood. On the one hand, the tropism of cancer cells to selected organs pinpoints the involvement of organ-specific factors that direct metastasis [1, 2]. On the other hand, factors that promote the formation of a pre-metastatic niche for metastasizing tumor cells provide a favorable growth and survival environment. Several organ-derived soluble factors are involved in this process, including a repertoire of chemoattractants secreted at sites of future metastasis, the expression of adhesion molecules crucial for attaching circulating tumor cells to the endothelium in blood vessels at future metastatic sites, and proteolytic enzymes secreted by cancer cells that facilitate their migration in tissues [3–5].

The first step in the metastatic process is egress of cancer cells from the primary tumor site into the interstitial fluid, followed by their migration to the lymph and subsequently to blood vessels. Taking into consideration that cancer cells preferentially infiltrate lymph nodes and spread through the lymphatics, the unanswered question remains: What are the crucial factor(s) directing egress of tumor cells from the primary tumor and their penetration into the lymphatics, body cavities, and peripheral blood?.

Many factors have been proposed to direct the metastatic process, including chemokines, cytokines, growth factors, complement cascade cleavage fragments, eicosanoids, bioactive lipids, and even extracellular nucleotides [6–13]. However, the chemotactic activities of most of these factors for malignant cells have been demonstrated *in vitro* at hyperphysiological concentrations relative to their normal levels in the tissues [6–8].

It is well known that plasma and serum by themselves have pro-migratory activity [14, 15], but the potential factor(s) contained in plasma and serum that are responsible for this effect are not well characterized. Such activity is usually assigned to chemokines and growth factors; however, the measured concentrations of these factors show that they are present at very low concentrations, which does not explain the robust chemotactic responsiveness of tumor cells to serum, even if some additional effect of these factors are involved.

In our current study we employed plasma and serum at different concentrations (0–90%) as chemotactic factors for several cancer cell lines and compared their chemotactic activities to known chemoattractants, such as hepatocyte growth factor/scatter factor (HGF/SF) [6] and α-chemokine stromal-derived factor 1 (SDF-1) [7].

We provide evidence that vitronectin is the most potent pro-migratory factor in peripheral blood and that its activity is inhibited after binding to fibrinogen. We propose that, in diluted plasma or serum depleted of fibrinogen, vitronectin is freed from this inhibitory complex with fibrinogen and is responsible for the pro-migratory activity of cells. Moreover, as confirmed here, vitronectin exerts this effect by activating urokinase plasminogen activator receptor (uPAR). In summary, we propose a new explanation for the role of vitronectin in the preferentially egress of cancer cells from tumors, spreading through the lymphatics and metastasizing to body cavities, which are both low in fibrinogen.

## RESULTS

### A remarkable effect of diluted human plasma on the migration of cancer cells

**Figure 1:**
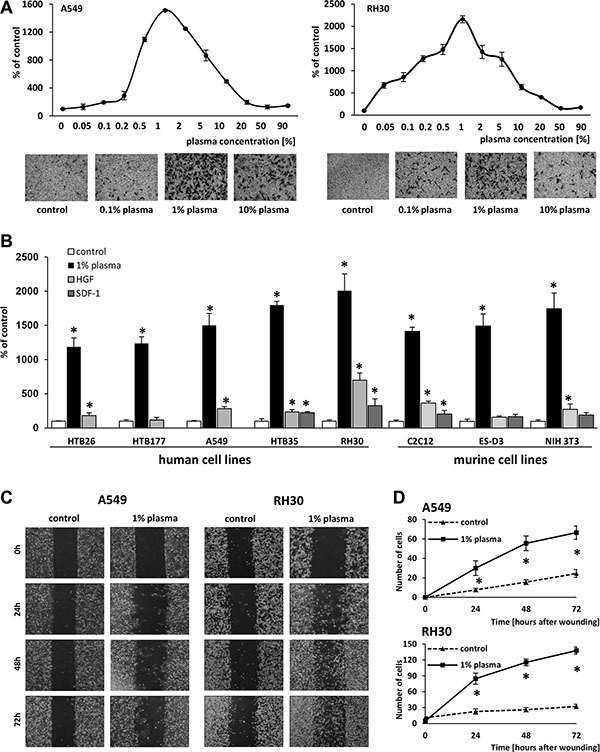
One-percent human plasma induces robust migration of various cell lines (Panel **A)** The dose-dependent effect of human plasma on the migration of A549 and RH30 cells, with sample images of stained cells from the Transwell inserts (lower panels) (Panel **B)** Migration of various human and murine cancer cell lines across Transwell membranes in response to 1% human plasma, HGF (10 ng/ml), or SDF-1 (300 ng/ml). *p < 0.05. (Panels **C** and **D**) The migration of A549 and RH30 cells in a wound healing assay. (Panel C**)** Examples of images taken under a microscope at different time points. (Panel D) The number of cells present within the wound at different time points. The experiment was performed twice, and the cells were counted in at least six areas. *p < 0.05.

Analyzing the migratory response of lung adenocarcinoma A549 cells (Figure 1A left panel) and rhabdomyosarcoma RH30 cells (Figure 1A right panel) in response to different plasma concentrations, we found to our surprise that the most robust response was to diluted (~1%) human plasma. Moreover, the chemotactic responsiveness of the cells decreased steeply at higher plasma concentrations.

Next, we analyzed whether a similar response could be observed for other cancer cell lines. Figure 1B demonstrates that the response of different human cancer cell lines, including breast cancer (HTB26), lung cancer (HTB177 and A549), cervical carcinoma (HTB35), rhabdomyosarcoma (RH30), murine myoblastic sarcoma (C2C12), murine immortalized embryonic (ES-D3), and murine fibroblastic (NIH 3T3) cells, to 1% plasma was much higher than to SDF-1 or HGF, which are known chemoattractants for these cells and employed at supraphysiological concentrations.

The migratory effect of diluted (1%) plasma was subsequently confirmed for A549 and RH30 cells in a wound healing assay (Figure 1C, 1D). We also tested whether non-malignant cells respond to 1% plasma and found that murine clonogenic hematopoietic progenitors (Supplementary Figure 1A) as well as human malignant non-adherent monocytic cells (THP-1) (Supplementary Figure 1B) also respond to diluted plasma and serum.

### Diluted (1%) plasma activates intracellular signaling in a G protein-coupled receptor-dependent manner

**Figure 2:**
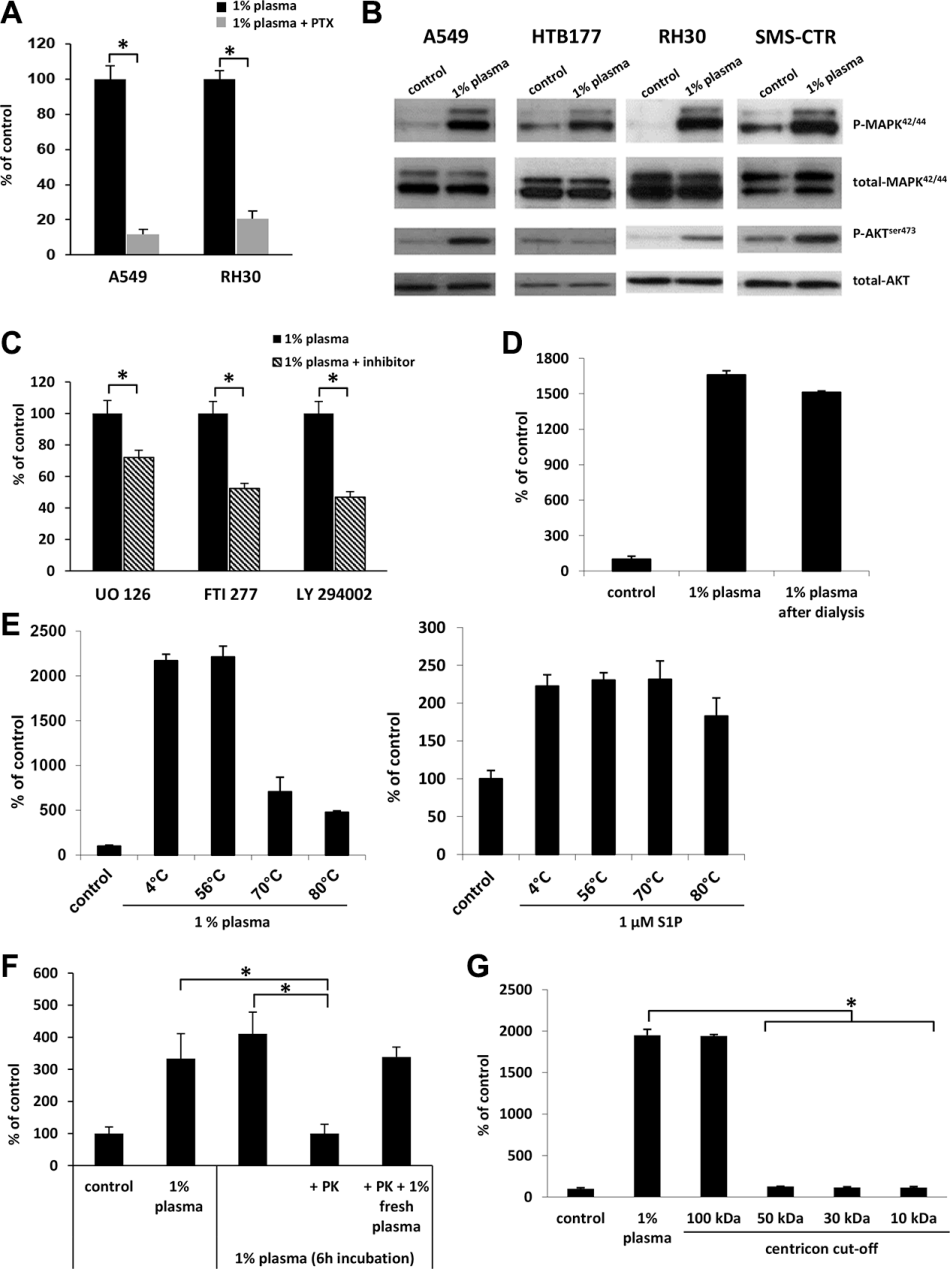
The factor(s) responsible for migration in response to 1% plasma are proteins that activate several intracellular pathway proteins in a G protein-coupled receptor-dependent manner (Panel **A**) The effect of pertusis toxin (PTX) on the migration of A549 and RH30 cells in response to 1% plasma. *p < 0.05. (Panel **B**) Phosphorylation of p42/44 MAPK and AKT in human cancer cell lines stimulated for 5 min with 1% plasma. The experiment was repeated twice with similar results, and a representative blot is shown. (Panel **C**) The effect of UO126, FTI277, and LY294002 signaling inhibitors on the migration of A549 cells in response to 1% plasma. *p < 0.05. (Panel **D**) The migration of A549 cells across Transwell membranes in response to 1% plasma or 1% plasma dialyzed over a 3.5-kDa-MWCO membrane. (Panel **E**) The temperature-dependence of 1% plasma preincubation on A549 cell migration. As a control, sphingosine-1-phosphate was used. *p < 0.05. (Panel **F**) The migration of A549 cells in response to 1% plasma preincubated with proteinase K (PK). To confirm the complete inhibition of PK by PMSF throughout the chemotaxis, a rescue experiment was performed in which 1% fresh plasma was added. *p < 0.05 (Panel **G**) The migration of A549 cells across Transwell membranes in response to different plasma fractions obtained by centrifugation in Centricon filters with different MWCOs.

To address whether the migratory effect of 1%-plasma is mediated through G_αI_ protein-coupled receptor(s), we analyzed the migration of A549 and RH30 cells in response to 1% plasma after pre-treatment of cells with pertussis toxin. Figure 2A shows a significant reduction in migration of cells in the presence of pertussis toxin when compared with control, untreated cells. Further analysis revealed that 1% human plasma stimulates phosphorylation of p42/44 MAPK and AKT in several human cell lines (Figure 2B). Finally, studies employing MEK1/2, H/K-Ras, and PI3K inhibitors (UO126, FTI277, and Ly294002, respectively) support the involvement of these signaling pathways in the chemotactic responsiveness of cancer cells to 1% plasma (Figure 2C).

### Evidence that the migration-enhancing factor(s) present in 1% plasma is a protein with molecular mass in the range 50–100 kDa

To characterize the molecule(s) in diluted plasma responsible for enhanced cell migration, we employed several complementary approaches. First, in order to eliminate small molecules, such as chemotactic bioactive lipids (e.g., sphingosine-1-phosphate, S1P; or ceramide-1-phosphate C1P) or extracellular nucleotides (e.g., ATP and UTP), we dialyzed dilute plasma through 3.5-kDa-MWCO membranes. Figure 2D demonstrates that dialysis did not affect the chemotactic activity of the diluted plasma, which suggests that molecules smaller than 3.5 kDa, (e.g., bioactive lipids or extracellular nucleotides) are not responsible for the observed pro-migratory activity of the diluted plasma.

This lack of change in chemotactic activity after dialysis suggested that the putative factor(s) has a peptide/protein structure. To confirm this, we first exposed human plasma to high temperature and, as expected, observed a decrease in its chemotactic activity (Figure 2E left panel). In a control experiment, high temperature did not affect the chemotactic responsiveness of A549 cells to the bioactive lipid S1P, as predicted (Figure 2E right panel). Next, the likelihood that the factor(s) present in diluted plasma is a protein or peptide was further confirmed by exposure of 1% plasma to proteinase K (Figure 2F). Finally, molecular filtration studies revealed that the putative chemotactic protein(s) has a size in the range 50–100 kD (Figure 2G).

### Differences in chemotactic responsiveness between diluted plasma and serum

**Figure 3:**
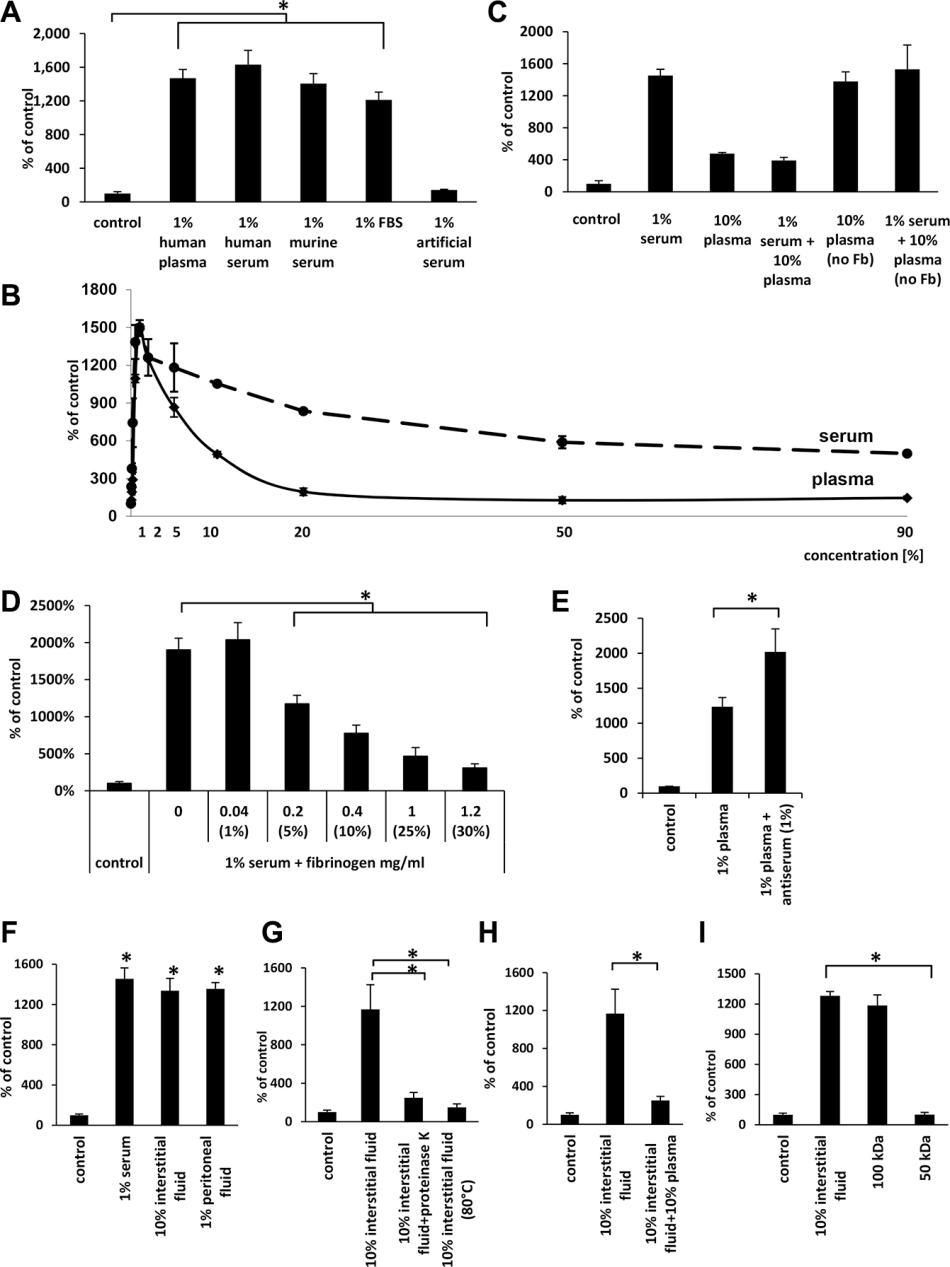
The effects of human plasma as well as different sera, interstitial fluids, and peritoneal fluids indicate that the factor(s) responsible for migration are present in different biological fluids and might be quenched by fibrinogen (Panel **A**) The effect of various 1% sera and human plasma on the migration of A549 cells. *p < 0.05. (Panel **B**) Differences in the dose-dependent migration of A549 in response to human plasma and serum. (Panel **C**) Migration of A549 cell lines in response to plasma and serum without fibrinogen (Fb). (Panel **D**) The inhibitory effect of fibrinogen (Fb) on the migration of A549 cells in response to 1% serum. The values in brackets represent the concentrations of plasma corresponding to each fibrinogen concentration. *p < 0.05. (Panel **E**) The effect of fibrinogen antiserum on the migration of A549 cells in response to 1% plasma. *p < 0.05. (Panel **F)** The effect of interstitial and peritoneal fluids on the migration of A549 cells. *p < 0.05. (Panel **G**) The migration of A549 cells across Transwell membranes in response to 10% interstitial fluid after proteinase K treatment (PK) or heat-inactivation (HI; 80°C). *p < 0.05. (Panel **H**) The inhibitory effect of 10% plasma on A549 cell migration in response to 10% interstitial fluid. (Panel **I)** The migration of A549 cells across Transwell membranes in response to different fractions of interstitial fluid obtained by centrifugation in Centricon filters with different molecular weight cut offs (MWCOs). *p < 0.05.

As shown in Figure 3A, we compared the migratory effect of 1% plasma and serum with 1% murine serum, 1% fetal bovine serum (FBS), and 1% artificial serum, and, except for artificial serum, we observed a similar migratory response, which suggests that a species-conserved protein(s) is responsible for this effect.

Next, we compared the pro-migratory effect of various dilutions of plasma and serum side-by-side. Figure 3B shows that, like plasma, the highest activity for serum was observed at 1% dilution. However, in contrast to serum, the pro-migratory effect of plasma significantly decreased at lower dilutions and reached a minimum at 20% plasma.

Since the major difference in protein composition between plasma and serum is the lack of fibrinogen and the lower level of coagulation factors, which are used up during the clotting of serum, we focused on the potential role of fibrinogen as a factor chaperoning the chemotactic activity of the 50–100-kD protein(s) present in plasma. To address this issue, we employed several complementary experimental strategies. Figure 3C shows that when we combined 1% serum with 10% plasma (which contains fibrinogen) in our migration assays, the chemotactic activity of the serum was significantly decreased. By contrast, when we employed 10% plasma from which fibrinogen, but not clotting factors, had been removed, it had similar activity as 1% serum and did not affect the migratory response of cancer cells if added to 1% serum.

To further probe this observation, we added to diluted (1%) serum increasing doses of purified fibrinogen and observed a dose-dependent inhibition of cell migration (Figure 3D). Moreover, when we added antiserum against fibrinogen to 1% plasma, we observed an increase in migration of A549 cells (Figure 3E). In control experiments addition of fibrinogen to another known chemoattractant for cancer cells that is SDF-1 did not affect cell migration, what indicates selectivity of fibrinogen in neutralizing investigated in our studies chemotactic factor (data not shown).

### Human interstitial and peritoneal fluids have similar cell migratory activity as diluted plasma and serum

Since a primary site of egress of tumor cells from primary tumor is the intercellular space, which is filled with interstitial fluid, we employed interstitial fluid harvested from normal patients in our cell migration assay. As shown in Figure 3F, both 10% interstitial fluid and 1% peritoneal fluid show similar activity to 1% serum. These concentrations of biological fluids employed in our experiments were normalized based on total protein content in particular samples. Moreover, the chemotactic activity of 10% interstitial fluid was inhibited by exposure to proteinase K or heat inactivation at 80°C (Figure 3G) and by addition of 10% plasma to 10% interstitial fluid (Figure 3H). Furthermore, molecular filtration studies revealed that this migratory activity in interstitial fluid is contained, as we observed for plasma, in the 50–100-kD protein fraction (Figure 3I).

### Evidence that diluted human plasma stimulates mainly random chemokinesis rather than directed chemotaxis of A549 cells

**Figure 4:**
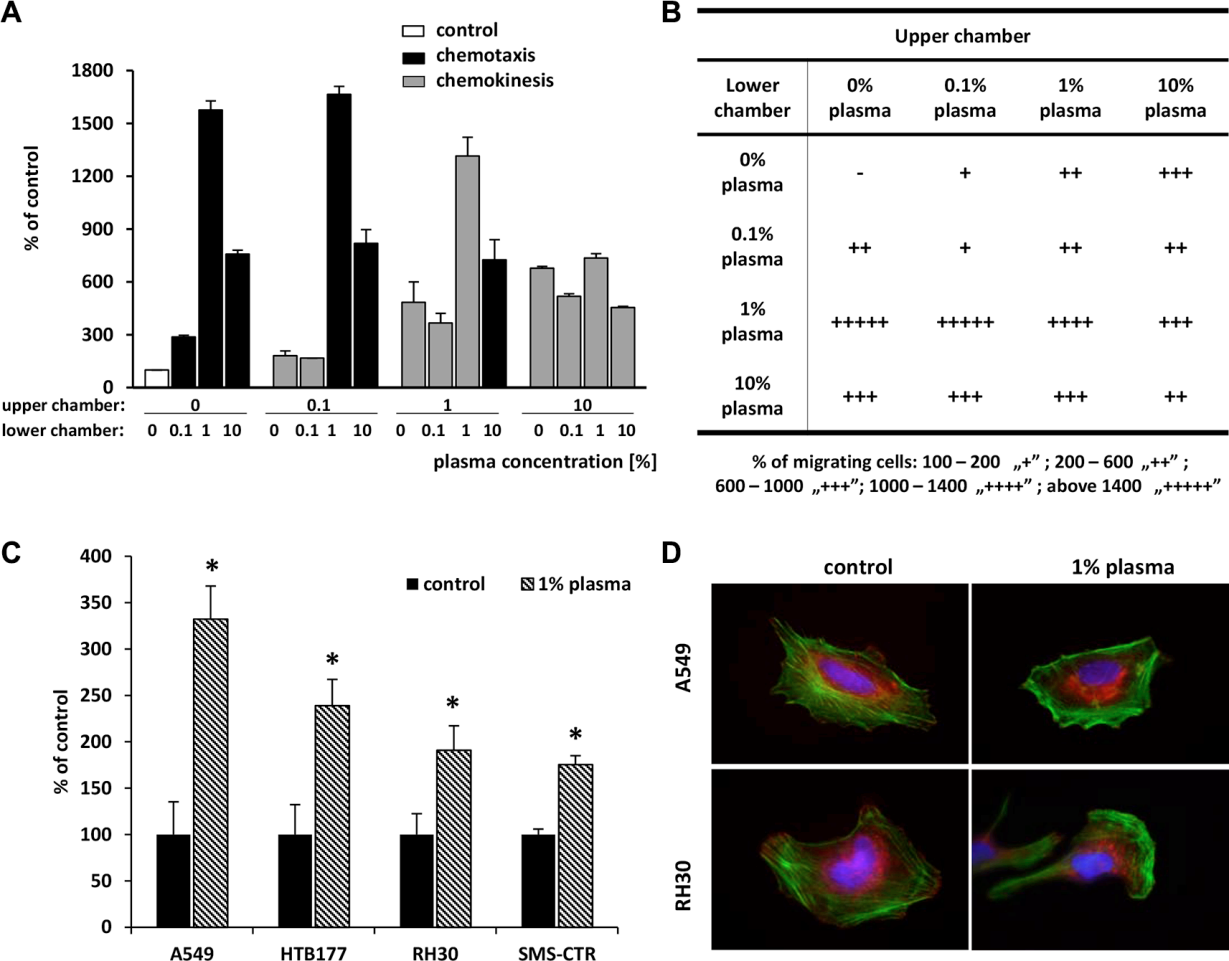
Highly diluted plasma induces mainly a chemokinetic response and affects adhesion, actin cytoskeleton organization, and proliferation (Panels **A** and **B**) Chemotaxis and chemokinesis analysis of A549 cell migration in the presence of different plasma concentrations in the upper and lower chambers. (Panel A) Graphic representation of checkerboard assay results. The black bars are for chemotaxis conditions, while the grey bars are for chemokinesis conditions. Panel B. The number of cells that migrated across Transwell membrane was determined and presented according to the legend. (Panel **C**) The adhesion of A549, RH30, SMS-CTR, and HTB177 cells to fibronectin after stimulation with 1% plasma for 5 min. *p < 0.05. (Panel **D**) Actin cytoskeleton organization of A549 and RH30 cells in medium alone and after 1-h stimulation with 1% plasma. F-actin in green, paxillin staining in red. Representative images are shown.

To determine whether the effect of human plasma on migration is due to directional (chemotaxis) or random (chemokinesis) motility, we performed a checkerboard assay using Transwell chambers (Figure 4A, 4B). We again observed that the highest number of cells that migrated in response to 1% human plasma in the lower chamber was also observed in the absence of a diluted plasma gradient—when equal concentrations of plasma were placed into the upper and lower chambers or when the concentration of plasma was higher in the upper chamber. This result indicates that diluted human plasma stimulates a chemokinetic rather than a chemotactic response.

Since cell adhesion plays an important role in tumor metastasis, we evaluated the influence of 1% plasma on adhesion of A549, HTB177, RH30, and SMS-CTR cells to fibronectin (Figure 4C) and found that 1% human plasma significantly increases adhesion of these cells in addition to chemokinesis.

Dynamic reorganization of the cytoskeleton is a prerequisite for cell adhesion and migration. Thus, to determine the effect of diluted (1%) plasma on the F-actin cytoskeleton, the A549, and RH30 cell lines were stimulated with 1% plasma, and F-actin fibers were visualized by Alexa Fluor 488–phalloidin staining. Figure 4D shows that in the absence of 1% plasma, cancer cells displayed well-developed bundles of F-actin, arranged in parallel to the long axis of the cells. By contrast, after exposure to 1% plasma, we observed redistribution of the actin cytoskeleton toward the leading edge of the cell. Furthermore, 1% plasma also altered the sub-cellular localization of paxillin. In control cells paxillin is located at focal adhesions, whereas in 1% plasma-treated cells it is translocated into the cytoplasm. This molecular redistribution provides additional support for the induction of a motility phenotype in malignant cells exposed to 1% plasma.

### The identification of protein(s) in diluted plasma responsible for the robust chemokinesis of cancer cells

**Figure 5:**
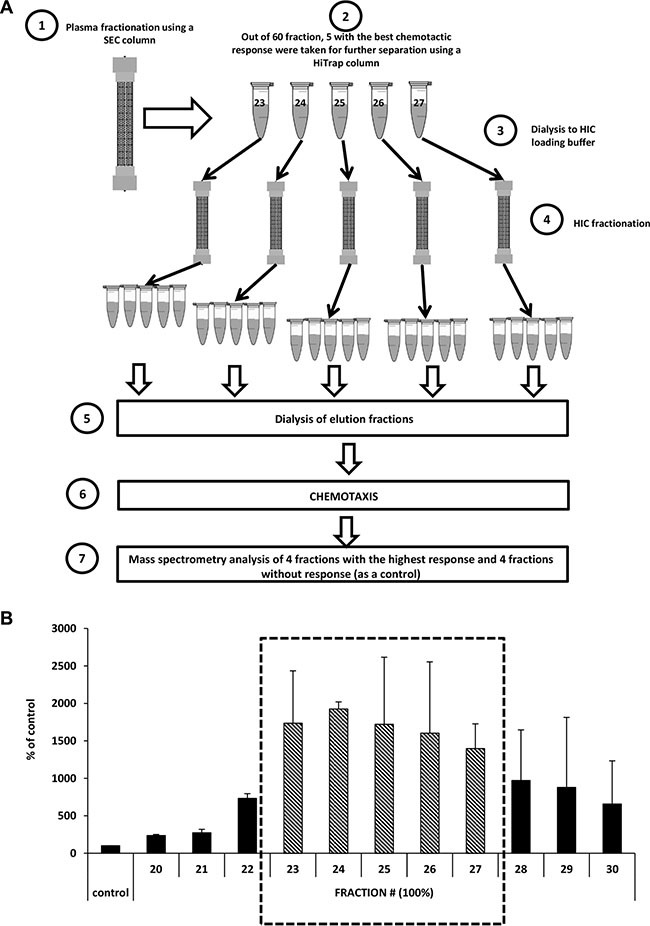
Identification of the factor(s) responsible for the migration of cells in response to highly diluted plasma (Panel **A**) Scheme of the size-exclusion chromatography (SEC) and hydrophobic interaction chromatography (HIC) experiments. (Panel **B**) Combined results of chemotaxis in response to different fractions after SEC separation (the average of two different fractionation runs).

To identify the protein(s) responsible for the robust chemokinesis observed in response to 1% plasma, we combined two chromatography-based plasma fractionation methods with chemotaxis and mass spectrometry characterization (Figure 5A). In the first step, whole human plasma was applied to a preparative scale size-exclusion chromatography (SEC) column and 60 fractions were collected encompassing the entire molecular weight range of the plasma constituents. Based on the results shown in Figure 2G, from these 60 plasma fractions in step 2 we selected 11 fractions that contained proteins in the size range of 50–100 kDa and subsequently selected for step 3 fractions #23–27, which had the highest migratory activity (Figure 5B). In step 4 of the fractionation procedure (Figure 5A), we performed hydrophobic interaction chromatography (HIC) and collected 65 samples. In step 5, we dialyzed the fractions obtained against PBS, and their pro-migratory activity was studied in Transwell migration assays (step 6). Based on this migratory activity, we finally selected four fractions that strongly induced migration of A549 cells as well as four fractions for which we did not observe increased migration (which were used as a control). These fractions were then subjected to mass spectrometry (MS) analysis (step 7). From the MS-identified proteins (Supplementary Figure 2), we selected three that were highly enriched in fractions with high pro-migratory activity compared with control fractions without chemokinetic activity. Two of these, vitronectin and low-molecular-weight kininogens (LMWK), were previously described as factors that affect cell migration. A third protein, hemopexin, was selected from the list based on structural similarities to vitronectin.

### Soluble vitronectin is the main protein present in human plasma that stimulates the migration of cells, and its activity can be quenched by fibrinogen

**Figure 6:**
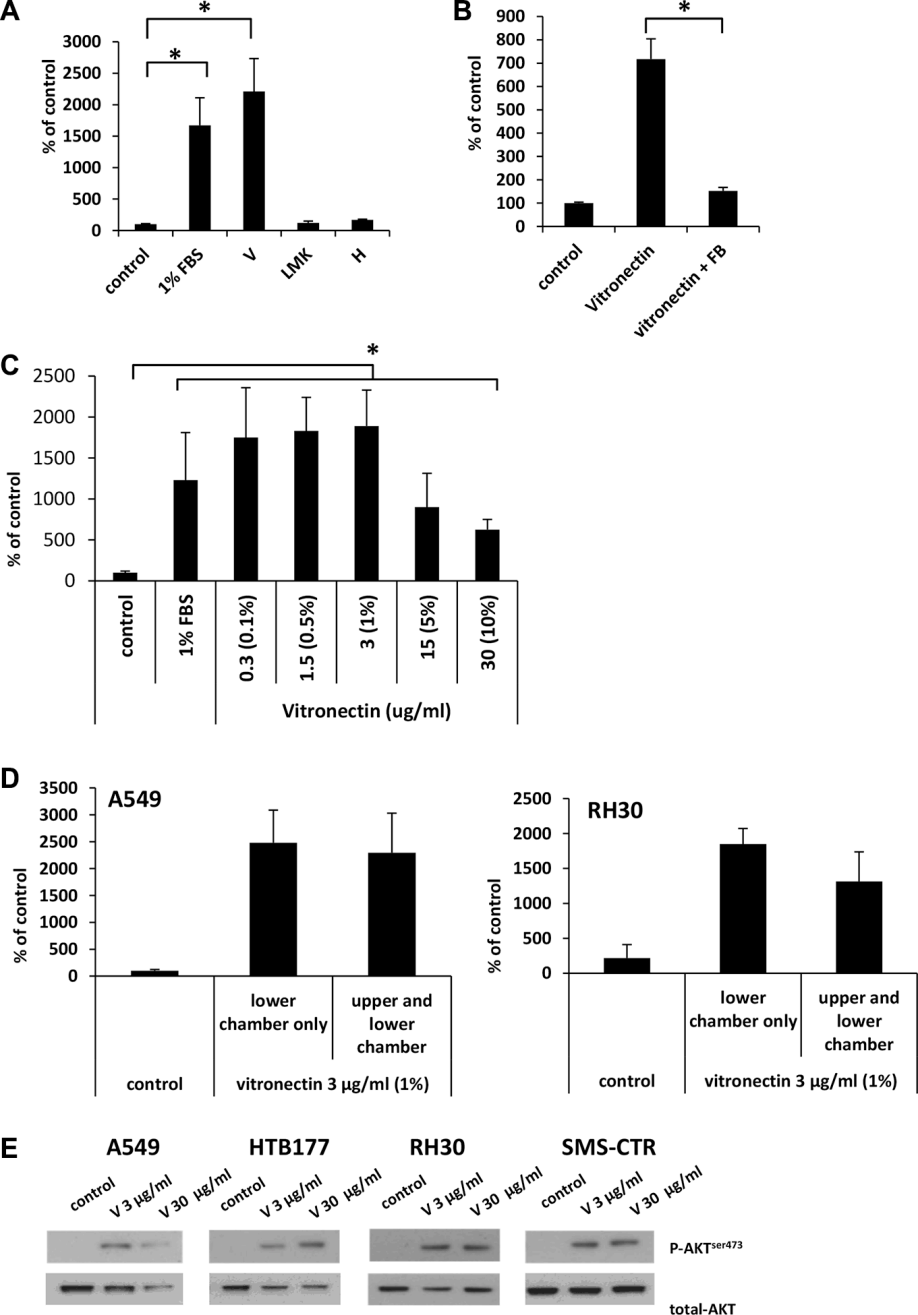
Vitronectin is the main factor responsible for the robust migration of cells in response to 1% plasma (Panel **A**) The migration of A549 cells across Transwell membranes in response to vitronectin (V), low molecular kininogens (LMK), and hemopexin (H). *p < 0.05. (Panel **B**) The inhibitory effect of fibrinogen (FB) on the migration of A549 cells in response to vitronectin. (Panel **C)** Dose-dependent migration of A549 cells in response to vitronectin. The values in brackets correspond to the dilution of plasma at which the same concentration of vitronectin is detected. *p < 0.05. (Panel **D**) Analysis of chemotaxis and chemokinesis of A549 and RH30 cell migration in the presence (vitronectin in the lower chamber only) or the absence (vitronectin present in both chambers) of a vitronectin gradient. (Panel **E**) Phosphorylation of AKT in human cancer cell lines stimulated for 5 min by vitronectin. The experiment was repeated twice with similar results, and representative results are shown.

Based on the MS results, we tested whether vitronectin, LMWK, and hemopexin have pro-migratory activity against A549 cells. We found that vitronectin, at a concentration corresponding to that present in 1% human plasma (3 μg/ml) [16], highly stimulates the migration of cells at a similar or even stronger degree than 1% serum (Figure 6A). At the same time, neither LMWK nor hemopexin were able to significantly stimulate the migration of cells. We obtained a similar responsiveness for other cell lines (HTB177 and RH30), which were selected based on the experiments performed in Figure 1B (data not shown).

To test whether the pro-migratory activity of vitronectin, like 1% plasma, can be quenched by fibrinogen, we added fibrinogen to vitronectin and observed an inhibition of cell migration (Figure 6B). Next, we tested the dose-dependent migration response of A549 cells to vitronectin, and we found that the highest response was observed for concentrations of vitronectin corresponding to those present in 0.1–1% plasma (Figure 6C).

Since 1% plasma was shown to have mostly chemokinetic activity (Figure 4A, 4B), we therefore tested whether purified vitronectin exhibits a similar effect. As can be seen in Figure 6D, both A549 (left panel) and RH30 cells (right panel) respond by chemokinesis to vitronectin. We also tested whether the analyzed cell lines express vitronectin receptors. Supplementary Figure 3 shows that all cells employed in our studies express the urokinase (uPAR) and integrin α_5_β_1_ receptors. Another integrin-type receptor, α_v_β_3_, was expressed by two out of four cell lines. More importantly, vitronectin receptors are functional, since stimulation of cell lines with vitronectin induced AKT phosphorylation (Figure 6E).

**Figure 7:**
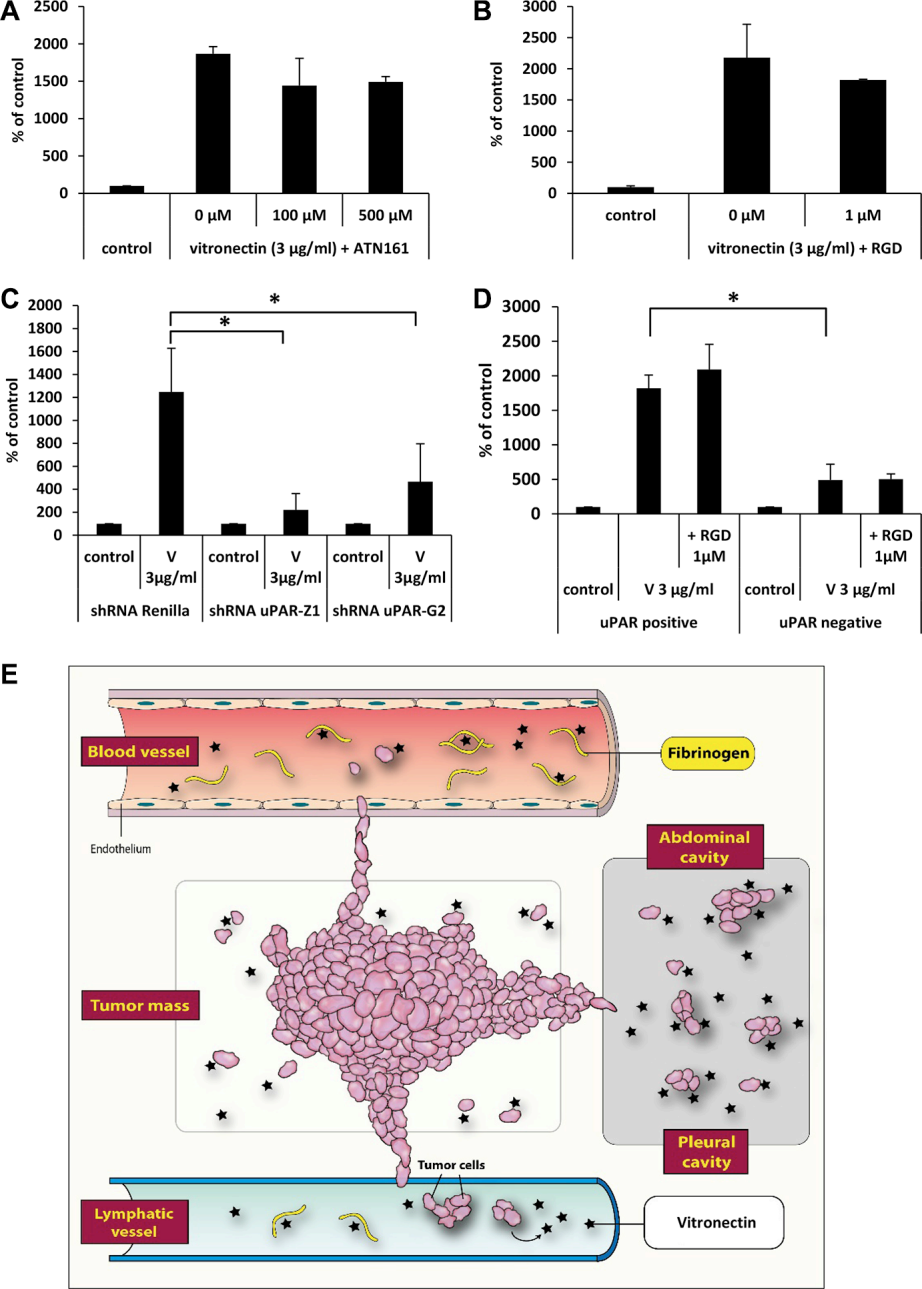
The effect of vitronectin on the migration of cancer cells The effect of integrin receptor antagonists ATN161 (Panel **A**) and RGD (Panel **B**) on A549 cell migration in response to vitronectin. *p < 0.05. (Panel **C**) The effect of downregulation of the uPAR receptor on the response of cells to vitronectin. *p < 0.05. (Panel **D**) Differences in response to vitronectin of two sorted subpopulations of A549 cells, with a low or high level of uPAR receptor expression. (Panel **E**) Scheme indicating the role that vitronectin and fibrinogen play in the metastasis of cancer cells.

Finally, to identify the receptors involved in the observed phenomena we employed the integrin receptor antagonists ATN161 and RGD (Figure 7A, 7B) and did not observe any effect of integrin receptor inhibition on cell migration. Therefore, we focused our attention on the potential role of uPAR. To address the role of this receptor, we transfected A549 cells with plasmids coding two different shRNAs against uPAR or with control plasmid (shRNA against *Renilla*) (Figure 7C). We also sorted A549 cells into populations enriched for high uPAR expression and for very low uPAR expression (Figure 7D) and tested their responsiveness to vitronectin.

We found that downregulation of the uPAR receptor by 50% (uPAR sh-Z1) or 30% (uPAR sh-G2), based on FACS analysis (data not shown), decreased the migratory response of cells to vitronectin (Figure 7C). Similarly, cells with lower uPAR expression showed a weaker response to vitronectin (Figure 7D), and this response was not affected by addition of the integrin inhibitor RGD. Our p42/44 MAPK and AKT signaling experiments and specific kinase inhibitor studies revealed involvement of kinase signaling pathways in cell migration. Since migration was dependent on presence of functional uPAR receptor and not integrin receptors expressed on cancer cells, our results indicate a leading role of signaling from activated uPAR receptor expressed on cancer cells in metastasis.

## DISCUSSION

The most important finding of this report is the pro-migratory effect of vitronectin, which directs metastasis of cancer cells to low-fibrinogen environments (e.g., lymphatics or body cavities) in a uPAR-mediated manner. We also provided evidence that vitronectin not only plays a role in cell adhesion but is a very potent chemokinetic factor that, if freed from the chaperone complex with fibrinogen, increases the migration of cancer cells to a much higher degree than other known chemoattractants.

Cancer metastasis is an unsolved clinical problem, and the mechanisms behind this serious complication are still not completely understood [3, 17]. It is known that several chemokines, growth factors, cytokines, bioactive lipids, extracellular nucleotides, and even H^+^ protons promote directed chemotactic and random chemokinetic migration of cancer cells [6–13]. Also involved in this process of facilitating migration are proteolytic enzymes released by cancer cells and adhesion molecules expressed on their surface. In addition, other mechanisms are also involved, such as the epithelial–mesenchymal transition [18].

In studying the factors involved in cancer metastasis, we observed a surprisingly high pro-migratory activity induced by diluted (1%) plasma or serum. This activity surpassed by several fold the effects of known chemotractants for cancer cells, such as SDF-1 [7] or HGF/SF [6]. Vitronectin was four-fold more effective than these known chemoattractants, which were employed in our assays at supraphysiological doses. Interestingly, this activity was quenched by increasing the concentration of fibrinogen in plasma or serum preparations. We followed up on this intriguing observation, and by employing several complementary strategies based on molecular filtration, chromatography-based plasma fractionation and MS analysis, we identified this factor as vitronectin.

Vitronectin belongs to the hemopexin family of glycoproteins and is found at high levels in peripheral blood, extracellular matrix, and bone [19]. It has been demonstrated to play an important role in the adhesion of cancer cells to integrins. Specifically, vitronectin has been shown to promote invasiveness of ovarian cancer cells and their spread in the peritoneum [20, 21]. The mechanisms responsible are related to MMP-2-mediated cleavage of vitronectin and fibronectin to generate fragments that interact with integrin receptors α_5_β_1_ and α_v_β_3_, which promote the adhesion of ovarian cancer cells lining the peritoneal cavity [22]. Molecular analysis of the vitronectin structure reveals that it contains three structural domains: i) an N-terminal somatomedin B domain, which binds to plasminogen activator inhibitor 1 (PAI-1); ii) a central domain containing hemopexin homology; and iii) a C-terminal domain that also has hemopexin homology [23]. Vitronectin also contains an RGD domain that binds to α_5_β_1_ and α_v_β_3_ integrin and the somatomedin B domain that binds to urokinase receptor (uPAR, also known as CD87). The interaction of vitronectin with uPAR has been proposed to be involved in cell migration [24]. Our results presented in this work demonstrate that vitronectin, by engaging the uPAR receptor, is a very potent chemokinetic factor for both cancer cells and normal cells.

What is most important is that the pro-migratory activity of vitronectin is quenched by fibrinogen. This soluble fibrinogen protein has been proposed to play a role in cancer metastasis, as it is a precursor of fibrin mesh formation, which may trap circulating cancer cells [25–27]. Here we provide evidence for its novel role as a chaperone, preventing vitronectin availability to engage with the uPAR expressed on cancer cells. Therefore, fibrinogen in this setting prevents vitronectin's pro-migratory effect. In support of an affinity between vitronectin and fibrinogen, the incorporation of vitronectin into fibrin clots has been reported [28] and, as reported in the literature, it is a significant problem to purify fibrinogen from vitronectin to obtain purified fibrinogen [28]. Since, only ~ 10% of total fibrinogen called *γA/γ′* fibrinogen is involved *in vitro* nectin binding, further studies with this fraction of fibrinogen [28] would be helpful to identify vitronectin binding site, what will be crucial do develop potential soluble vitronectin inhibitors.

Our results show that fibrinogen is important for chaperoning cell migration-promoting vitronectin. This complex seems to be destabilized when plasma is diluted, and the dissociation constant (Kd) regulating the interaction between fibrinogen and vitronectin promotes their dissociation in diluted plasma. With increasing plasma concentration, this effect is lost, and vitronectin again becomes chaperoned by fibrinogen. In contrast to plasma, more free vitronectin is present in fibrinogen-depleted serum.

This phenomenon and the mechanism described here have an important implication for the metastasis of cancer cells to environments with low fibrinogen concentrations. First, it most likely plays an important role *in vivo* in the egress of cells from a primary tumor into the interstitial fluid, where vitronectin is present and not chaperoned by fibrinogen. This egress is mediated by the random chemokinetic effect of vitronectin on cancer cells that leave an expanding tumor. Next, since the concentration of fibrinogen is low in lymph, this effect may explain the preferential migration of cancer cells into lymphatics and the subsequent infiltration of lymph nodes. Finally, cancer cells may also preferentially migrate into body cavities, where again the fibrinogen concentration is very low. As mentioned above, this latter phenomenon may explain the migration of ovarian cancer cells into peritoneal ascites [29]. All of these pro-metastatic effects of the vitronectin–fibrinogen–uPAR axis are summarized in Figure 7E. As mentioned above in addition to fibrinogen mediated effects reported herein, fibrinogen is also known to increase metastasis by trapping and immobilizing cancer cells in fibrin mesh network at site of future metastasis [25–27].

It is worth mentioning that an important metastasis-promoting mechanism also takes place in response to radio-chemotherapy, in which several pro-migratory factors are released from damaged cells [9–11, 30, 31]. These factors may promote metastasis by chemoattracting cancer cells that survived the initial treatment [9–11, 30]. Of note, we have already observed that the concentration of vitronectin increases in tissues irradiated or exposed to chemotherapeutic agents (manuscript in preparation). Thus, free vitronectin enriched in organs damaged after radio-chemotherapeutic treatment may provide or support an attractive gradient for malignant cells circulating in peripheral blood.

In conclusion, we identified a novel mechanism that regulates the migration of cancer cells, and we provided a novel explanation for why cancer cells preferentially migrate to lymphatics and body cavities. We also reported that uPAR is a crucial receptor for vitronectin after it is freed from an inhibitory complex with fibrinogen. This observation provides justification for the development of specific and more efficient uPAR inhibitors that could be employed as anti-metastatic drugs. Finally, vitronectin is not only a ligand for pro-adhesive integrins on the surface of cancer cells but is also a very potent chemokinetic factor.

## MATERIALS AND METHODS

### Cell lines

SMS-CTR, C2C12 NIH 3T3, and ES-D3 cells were cultured in Dulbecco's modified Eagle's medium (DMEM) containing 10% (or 15% for ES-D3) fetal bovine serum (FBS), 100 U/ml penicillin, and 10 μg/ml streptomycin. The medium for ES-D3 was additionally supplemented with leukemia inhibitory factor (LIF, 5 ng/mL; Shenandoah; Warwick, PA). Murine bone marrow-derived mesenchymal stromal cells (mBMMSC) cells were grown in DMEM supplemented with 10% horse serum, 10% FBS, and antibiotics (100 U/ml penicillin and 10 μg/ml streptomycin). HTB26, A549, HTB177, HTB35, and RH30 cells were maintained in RPMI 1640 medium containing 10% FBS, 100 U/ml penicillin, and 10 μg/ml streptomycin. All cells were grown at 37°C, 5% CO_2_, 95% humidity. All cell lines were either bought from American Type Culture Collection (ATCC) or were gifts from Dr. Peter Houghton, World Children's Cancer Center, Columbus, OH and Prof. Fred Bar, National Cancer Institute, Bethesda, MD and authenticated by STR analysis.

### Chemotaxis assay

Chemotaxis assays were performed as described [10, 31, 32] In some experiments the cells were pretreated with the inhibitors: pertussis toxin (PTX, 1 μg/ml) for 15 min or UO126 (1 μM), FTI277 (10 μM), LY294002 (100 ng/ml), ATN161 (100–500 μM) for 30 min or RGD (1 μM) for 60 min at 37°C. Inhibitors were also added to the lower chambers and were present throughout the experiment.

### Plasma modifications

Molecular-weight cut off (MWCO) plasma fractions were obtained by centrifugation of plasma in a Centricon Ultracel YM (Millipore) with different MWCOs (10, 30, 50, or 100 kDa). Only the flow-through fractions were used for experiments. Dialyzed plasma was obtained by dialysis of plasma samples against PBS (without calcium and magnesium, HyClone, Logan, UT) in a Slide-A-Lyzer dialysis cassette with a 3,500-Da MWCO (ThermoFisher Scientific, Waltham MA) and used for experiments. Plasma without proteins was obtained by a 4-h incubation of a 10% plasma sample (in PBS) at RT with proteinase K at a final concentration of 6 mAU/ml. Next, 10 μl of plasma was analyzed on a polyacrylamide gel stained with Coomassie Brilliant Blue R to confirm complete digestion of protein. The potential negative effect of proteinase K itself on cells was limited by adding 1 mM phenylmethyl sulfonyl fluoride (PMSF) into the sample before chemotaxis assay.

Fibrinogen-free plasma was obtained by adding 0.5 ml of a saturated solution of Na_2_SO_4_ into 2 ml of plasma to achieve 20% saturation. Following incubation for 60 min at 4°C, the mixture was centrifuged (10 min, 3824 × g) and the supernatant was dialyzed against PBS (10 mM, pH 6.5) for 24 h. The salting out efficiency was estimated by electrophoresis on an agarose gel.

### Plasma fractionation and mass spectrometry analysis

Plasma samples were size-fractionated using a HiLoad 16/600 Superdex 200 prep grade gel filtration column (GE Healthcare Biosciences, Piscataway, NJ) equilibrated in 1.7 mM KH_2_PO_4_, 8.3 mM K_2_HPO_4_, 150 mM NaCl, pH 7.5 employing a Waters HPLC system (Waters 2998 Photodiode Array Detector and Waters 600 Pump, Waters Corporation; Milford, MA) operated at room temperature. Undiluted whole plasma samples were filtered to remove particulates using Spin-X centrifuge tube filters (0.45 μm cellulose acetate; Corning Incorporated; Corning, NY). Samples of ~1 mL were injected and eluted at a flow rate of 0.5 mL/min at room temperature. Sixty fractions of ~1.75 mL were collected after a 70-min delay to encompass the entire elution profile. Fractions exhibiting a high chemotactic response were selected for additional chromatographic separation using a 1-mL HiTrap Butyl Sepharose Fast Flow hydrophobic column (GE Healthcare Biosciences; Piscataway, NJ), employing an ÄKTApurifier FPLC system (GE Healthcare Biosciences; Piscataway, NJ) at a flow rate of 1 mL/min at room temperature. Fractions were dialyzed using Slide-A-Lyzer dialysis cassettes (MWCO 3,500 Da; 0.5–3 mL; Pierce; Rockford, IL) against a 500-fold excess of 18.2 mM NaH_2_PO_4_, 31.8 mM Na_2_HPO_4_, 1 M (NH_4_)_2_SO_4_, pH 7.0 (buffer A) for 24 hours at 4°C, with three buffer changes, after 3 h, 4.5 h, and 14.5 h. The column was prepared by washing with 20 column volumes of deionized water, followed by 5 column volumes of 18.2 mM NaH_2_PO_4_, 31.8 mM Na_2_HPO_4_, 10 mM (NH_4_)_2_SO_4_, pH 7.0 (buffer B), then equilibrated in 25 column volumes of buffer A. Fractions dialyzed against buffer A were applied to the column, the column was washed with 20 column volumes of buffer A, and then eluted with a 20-column-volume linear gradient from 1 M (100% buffer A) to 25 mM (NH_4_)_2_SO_4_ (100% buffer B). The column was regenerated between samples by washing with 5 column volumes of deionized water followed by 5 column volumes of buffer A and then equilibrated in 25 column volumes of buffer A. Fractions of ~1 mL, collected during the sample wash and gradient elution steps, were analyzed for a chemotactic response.

After gel filtration, the fractions were used for chemotaxis without modification. Fractions obtained after passing through the HiTrap Butyl Sepharose Fast Flow hydrophobic column but before chemotaxis were dialyzed using Slide-A-Lyzer dialysis cassettes (MWCO 3,500 Da; 0.5–3 mL; Pierce) against PBS (pH 7.4, Sigma St. Louis, MO).

### Mass spectrometry analysis

Sample Preparation: Isolated fractions were digested using a filter-aided sample preparation (FASP) protocol. With this method the fractions were loaded into a YM-10 10,000Da molecular weight cutoff device, rinsed twice with 0.1M Tris-HCl (pH 8.5) and then digested overnight at 37°C with 20 ng sequencing grade modified trypsin in 100 μL of 1 M urea/0.1M Tris-HCl pH 8.5. This digest was filtered using the YM-10 device and the digests analyzed by a proteomic approach as described below. Samples were dried by speed vacuum centrifugation, resolubilized using 2% v/v acetonitrile/0.1% v/v formic acid, desalted and concentrated using C18 PROTOTM, 300Å Ultra MicroSpin Column (The Nest Group, Inc., Southborough, MA, USA) according to standard protocol. After clean up the samples were dried and redissolved in 40 μL 2% v/v acetonitrile/0.1% v/v formic acid. The absorbance of the redissolved sample was read at 205 nm using a NanoDrop 2000 and peptide values estimated. Sample volumes were adjusted to normalize estimated peptide concentrations to 0.25 μg/μL with 2% v/v acetonitrile / 0.1% v/v formic acid.

### LCMS data collection

Hi-Trap fraction digests (0.5 μg) were separated ona Dionex Acclaim PepMap 100 75 uM × 2 cm nanoViper (C18, 3 μm, 100Å) trap and Dionex Acclaim PepMap RSLC 50 uM × 15 cm nanoViper (C18, 2 μm, 100Å) separating columns. An EASY n-LC (Thermo) UHPLC system was used with buffer A = 2% v/v acetonitrile /0.1% v/v formic acid and buffer B = 80% v/v acetonitrile /0.1% v/v formic acid as mobile phases. Following injection of the sample onto the trap, separation was accomplished with an 80 min linear gradient from 0% B to 50% B, followed by a 5 min linear gradient from 50% B to 95% B, and lastly a 5min wash with 95% B. A 40 mm stainless steel emitter (Thermo) was coupled to the outlet of the separating column. A Nanospray Flex source (Thermo) was used to position the end of the emitter near the ion transfer capillary of the mass spectrometer. The ion transfer capillary temperature of the mass spectrometer was set at 225°C, and the spray voltage was set at 1.6 kV. Other chromatography settings are provided as Supplemental Information.

An Orbitrap Elite–ETD mass spectrometer (Thermo) was used to collect data from the LC eluate. An Nth Order Double Play with ETD Decision Tree method was created in Xcalibur v2.2. Scan event one of the method obtained an FTMS MS1 scan (normal mass range; 240,000 resolution, full scan type, positive polarity, profile data type) for the range 300–2000 m/z. Scan event two obtained ITMS MS2 scans (normal mass range, rapid scan rate, centroid data type) on up to twenty peaks that had a minimum signal threshold of 5,000 counts from scan event one. A decision tree was used to determine whether CID or ETD activation was used. An ETD scan was triggered if any of the following held: an ion had charge state 3 and m/z less than 650, an ion had charge state 4 and m/z less than 900, an ion had charge state 5 and m/z less than 950, or an ion had charge state greater than 5; a CID scan was triggered in all other cases. The lock mass option was enabled (0% lock mass abundance) using the 371.101236 m/z polysiloxane peak as an internal calibrant. Other method settings are provided as Supplemental Information.

### Data analysis

Proteome Discoverer v2.0.0.802 (Thermo) was used to analyze the data collected by the mass spectrometer. The database used in SequestHT searches was the 7/7/2015 version of the UniprotKB Homo sapiens reference proteome canonical and isoform sequences with the nonhuman sequences from the 1/1/2012 version of thegpm.org cRAP database appended to it.

The Proteome Discover analysis and consensus workflows are included in the Supplemental Information. The analysis workflow allows for extraction of MS2 scan data from the Xcalibur RAW file and searches of CID and ETD MS2 scans in SequestHT (.msf extension). In order to estimate the false discovery rate, a Target Decoy PSM Validator node was included in the Proteome Discoverer workflow. The consensus workflow allows for collection of the data from an msf file into a result file that can be viewed in Proteome Discoverer (.pdResult extension).

The study files from Proteome Discoverer were loaded into Scaffold Q+S v4.4.5 (Proteome Software, Portland, OR, USA). The false discovery rate for peptides was calculated using the Scaffold Local FDR algorithm. Protein probabilities were calculated using the Protein Prophet algorithm. Proteins were grouped by Scaffold protein cluster analysis to satisfy the parsimony principle. The results were annotated with human gene ontology information from the Gene Ontology Annotations Database (ftp.ebi.ac.uk).

### *In vitro* wound healing assay

Cells were seeded in a 40-mm dish, and 24 h later, a scratch through a monolayer of cells was made with a 0.2-ml pipette tip. Cell debris was removed, and a new aliquot of medium with 1% plasma or 1% artificial serum [33] was added. 24, 48, and 72 h after scratching, the cells were imaged using a microscope (Olympus America; Melville, NY) equipped with a CCD camera and the cells found in at least five different central areas of the scratch were counted.

### Cell proliferation

Cell proliferation was performed as described [9].

### Adhesion to fibronectin

Cells were made quiescent for 3 hours with 0.5% BSA in RPMI 1640 medium or DMEM before incubation with 1% plasma for 5 min. Subsequently, cell suspensions (5 × 10^3^/50 μl) were applied to 96-well plates covered with fibronectin (10 μg/ml) and incubated at 37°C for 10 min. The wells were coated with fibronectin overnight at 4°C and blocked with 0.5% BSA for 2 hours before the experiment. After incubation, the plates were washed three times to remove non-adherent cells, and the number of adherent cells was counted under an inverted microscope.

### Phosphorylation of intracellular pathway proteins

Western blots analysis was performed as described [9].

### Fluorescent staining of F-actin and paxillin

Cells were fixed in 3.5% paraformaldehyde for 20 min, permeabilized by 0.1% Triton X100, washed in PBS, pre-blocked with 2% BSA, and subsequently stained with paxillin (1:200, mouse monoclonal IgG, eBioscience) and phalloidin-Alexa Fluor 488 (1:400, Molecular Probes, Eugene, Oregon). Appropriate secondary Alexa Fluor 594 goat anti-mouse IgG antibodies were used (1:400, Molecular Probes, Eugene, Oregon). The nuclei were identified by staining with DAPI (Molecular Probes, Eugene, Oregon). The fluorescence images were collected with a confocal microscope (Olympus, Center Valley, PA).

### Flow cytometry

Cells were detached using CellStripper (Corning), followed by a 2-h incubation in appropriate medium with 2% FBS. Cells were stained with a mouse primary antibody against human integrin receptor α_5_β_1_ (1:25, Millipore, USA) for 30 min at 37°C. The cells were then washed and incubated with an Alexa Fluor 488 goat anti-mouse secondary antibody (1:100, Life Technologies). A murine phycoerythrin (PE)-conjugated anti-human CD87 antibody (1:20; clone VIM5, Biolegend) was used to analyze the uPAR receptor, and murine FITC-conjugated anti-human CD51/61 antibody (1:20; clone 2366, Biolegend) was used for staining of the human α_V_β_3_ integrin receptor. The cells were then analyzed using an LSR cell cytometer (BD Biosciences). Analysis of the data was performed using FlowJo 7.2.5 software (FLOWJO, Ashland, OR, USA). Unstained cells and cells incubated with isotype control antibodies were used as controls.

### Knockdown of S1PR1 with short hairpin RNA

In RNA interference (RNAi) experiments, the short hairpin RNA (shRNA)-generating plasmid pSUPER.retro. puro (Oligoengine) was used. The targeting base sequence for human uPAR was: 5′-GCTTGAAGATCACCAGCCTTA-3′ and 5′- GCACTCTCCTCTGGACCTAAA-3′. As a control, shRNA against *Renilla* was used, [34]. A549 cells were plated at 80% confluency and transfected with an shRNA vector using the TransIT-X2 System (Mirus) according to the manufacturer's protocol. Selection of pSuper.retro. puro–expressing cells was conducted by exposure of *in vitro* cultures to puromycin at a final concentration of 1.5 μg/mL for 6 days.

### Statistical analysis

All results are shown as mean ± standard deviation. Statistical analysis of the data was done using Student's *t-test* for unpaired samples, with *p* < 0.05 considered significant.

## SUPPLEMENTARY MATERIALS


