# Supplementary material for: Evidence that vitronectin is a potent migration-enhancing factor for cancer cells chaperoned by fibrinogen: a novel view of the metastasis of cancer cells to low-fibrinogen lymphatics and body cavities
Source: Oncotarget. 2016 Sep 13;7(43):69829–43. doi: 10.18632/oncotarget.12003 (PMC5342518; doi:10.18632/oncotarget.12003)
Supplement: Supplementary file 1 [file oncotarget-07-69829-s001.pdf]

Evidence that vitronectin is a potent migration-enhancing factor for cancer cells chaperoned by fibrinogen: a novel view of the metastasis of cancer cells to low-fibrinogen lymphatics and body cavities

Supplementary Materials

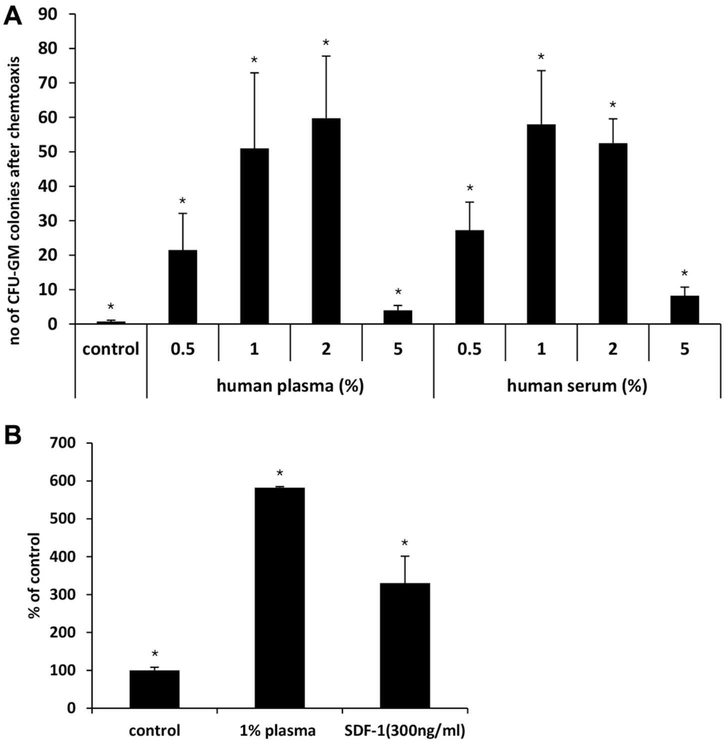

Supplementary Figure S1: One percent plasma induces robust migration of normal hematopoietic clonogenic progenitors and malignant non-adherent cells. (Panel **A**) The chemotactic responsiveness of clonogenic hematopoietic progenitor cells (colony-forming units of granulocyte-macrophages, CFU-GM) to low concentrations of human plasma or serum. Results are combined from two independent experiments. *$p > 0.05$. (Panel **B**) Migration of a human non-adherent leukemic cell line (THP-1) across Transwell membranes in response to 1% human plasma or SDF-1 (300 ng/ml). The chemotaxis assay was performed in duplicate with similar results. *$p < 0.05$.

	Protein name	Protein accession numbers	Database sources	Protein molecular weight (Da)	Protein identification probability
1	Hemopexin	sp|P02790|HEMO_HUMAN	HumanRef150707wC.fasta	51,676.50	100.00%
2	Alpha-2-HS-glycoprotein	sp|P02765|FETUA_HUMAN	HumanRef150707wC.fasta	39,323.40	100.00%
3	Vitronectin	sp|P04004|VTNC_HUMAN	HumanRef150707wC.fasta	54,306.10	100.00%
4	Plasminogen	sp|P00747|PLMN_HUMAN	HumanRef150707wC.fasta	90,567.40	100.00%
5	Isoform LMW of Kininogen-1	sp|P01042-2|KNG1_HUMAN	HumanRef150707wC.fasta	47,883.60	100.00%
6	Gelsolin	sp|P06396|GELS_HUMAN	HumanRef150707wC.fasta	85,697.80	100.00%
7	Afamin	sp|P43652|AFAM_HUMAN	HumanRef150707wC.fasta	69,070.10	100.00%
8	Alpha-1-antichymotrypsin	sp|P01011|AACT_HUMAN	HumanRef150707wC.fasta	47,653.00	97.60%
9	Coagulation factor XII	sp|P00748|FA12_HUMAN	HumanRef150707wC.fasta	67,790.60	100.00%
10	Heparin cofactor 2	sp|P05546|HEP2_HUMAN	HumanRef150707wC.fasta	57,072.90	100.00%
11	Angiotensinogen	sp|P01019|ANGT_HUMAN	HumanRef150707wC.fasta	53,154.80	100.00%
12	Complement factor H-related protein 1	sp|Q03591|FHR1_HUMAN,tr|B1AKG0|B1AKG0_HUMAN	HumanRef150707wC.fasta	37,650.40	100.00%
13	Isoform 2 of N-acetylmuramoyl-L-alanine amidase	sp|Q96PD5-2|PGRP2_HUMAN,sp|Q96PD5|PGRP2_HUMAN	HumanRef150707wC.fasta	62,217.90	100.00%
14	Glutathione peroxidase 3	sp|P22352|GPX3_HUMAN,tr|A0A087X1J7|A0A087X1J7_HUMAN	HumanRef150707wC.fasta	25,553.20	100.00%
15	Apolipoprotein A-I	sp|P02647|APOA1_HUMAN	HumanRef150707wC.fasta	30,778.50	100.00%

Supplementary Figure S2: Mass spectrometry (MS) analysis of plasma fractions. A list of proteins that were enriched in fractions with high chemotactic activity compared with fractions with very weak or no chemotactic activity.

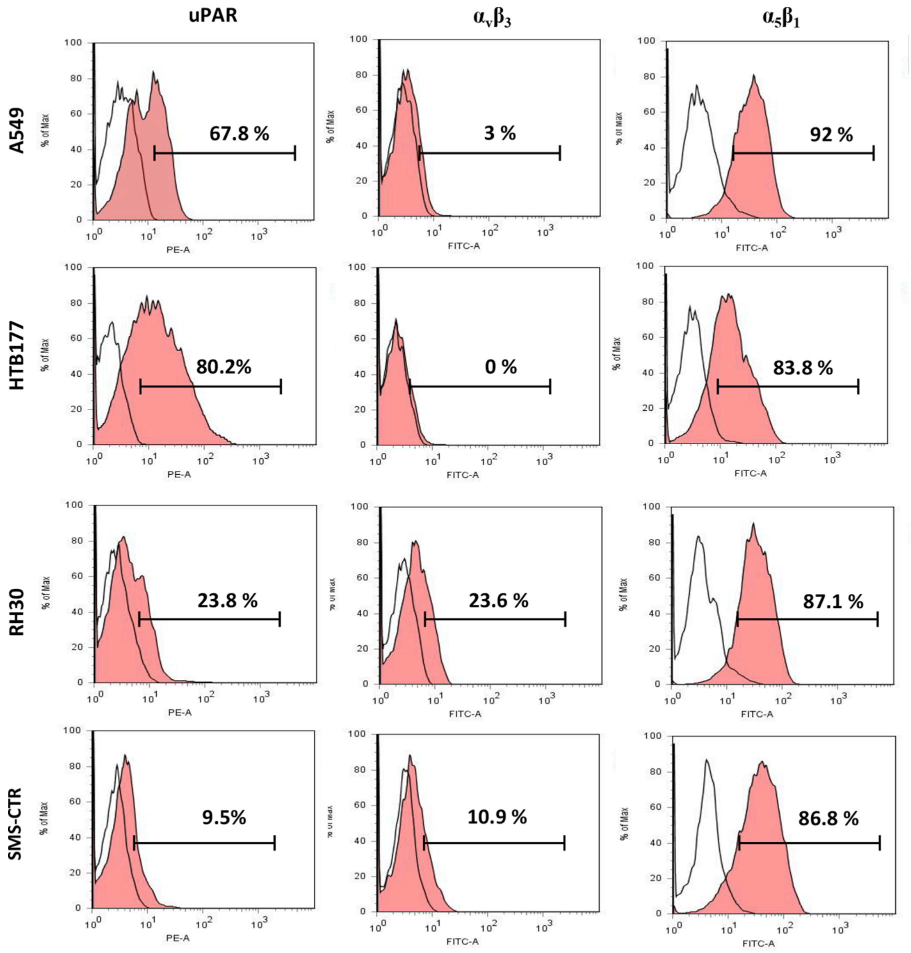

Supplementary Figure S3: The expression of vitronectin receptors in malignant cells. Flow cytometry analysis of the urokinase receptor (uPAR) and the two integrin receptors $\alpha_v\beta_3$ and $\alpha_5\beta_1$ in different cell lines.
